# Anxiety and Depression in Patients With Chronic Respiratory Diseases in the Fès-Meknès Region of Morocco

**DOI:** 10.7759/cureus.48349

**Published:** 2023-11-06

**Authors:** Nassiba Bahra, Bouchra Amara, Hind Bourkhime, Soukaina El Yaagoubi, Nada Otmani, Nabil Tachfouti, Mohamed Berraho, Mounia Serraj, Mohamed Chakib Benjelloun, Samira El Fakir

**Affiliations:** 1 Laboratory of Epidemiology, Clinical Research and Community Health, Faculty of Medicine and Pharmacy, Sidi Mohamed Ben Abdellah University, Fez, MAR; 2 Pulmonology Department, Hassan II University Hospital, Fez, MAR; 3 Epidemiology, Clinical Research and Public Health, Faculty of Medicine and Pharmacy, Sidi Mohamed Ben Abdellah University, Fez, MAR

**Keywords:** morocco, hads, depression, anxiety, chronic respiratory disease

## Abstract

Background

Chronic respiratory diseases (CRDs) are a major public health problem in Morocco. Several studies have shown that anxiety and depression are important comorbidities of CRDs and are often associated with CRDs. This study aimed to estimate the prevalence of depression and anxiety and identify their determinants in patients with CRDs.

Methodology

A cross-sectional study was conducted in the Pneumology Department at the Hassan II University Hospital in Fez in 2021. An anonymous questionnaire was used to collect sociodemographic, clinical, and therapeutic data. The Moroccan version of the Hospital Anxiety and Depression Scale (HADS) was used to measure depression and anxiety. A descriptive analysis was performed, followed by a bivariate analysis to investigate the association between anxiety and depression and other factors using tests appropriate to the types of variables studied. A p-value ≤0.05 was considered significant. Data entry was performed in Excel 2013 (Microsoft Corp., Redmond, WA, USA), and data analysis was done using SPSS software version 26 (IBM Corp., Armonk, NY, USA).

Results

The study included 209 patients, 50.7% (n = 106) of whom were female, with an average age of 57.84 ± 15.36 years. Chronic obstructive bronchopneumopathy was the most represented CRD (43.1%; n = 90), followed by asthma (32.2%; n = 67). The prevalence of depression and anxiety was 46.4% (n = 97) (95% confidence interval (CI) = 39.2-52.8) and 57.4 % (n = 120) (95% CI = 50.3-63.7), respectively. In the univariate analysis, depression was associated with the presence of dyspnea (51.3% vs. 32.7%; p = 0.018), the presence of asthenia (56.5% vs. 38.5%; p = 0.009), the use of oxygen therapy (66.7% vs. 42.7%; p = 0.015), and a higher number of hospitalizations (76.9% vs. 44.4%; p = 0.023). Moreover, 87.6% of patients with depression also had anxiety (p < 0.001). Anxiety was associated with a history of surgery (37.2% vs. 62.4%; p = 0.003) and with the presence of chronic obstructive pulmonary disease (66.7% vs. 50.4%; p = 0.019).

Conclusions

The results reveal the importance of screening for anxiety-depressive disorders in patients with CRDs and taking into account psychological aspects in the management of the disease to improve quality of life.

## Introduction

Chronic respiratory diseases (CRDs) are a public health problem that imposes a significant burden [1]. Worldwide, CRDs afflict hundreds of millions of individuals. Presently, there are 300 million individuals with asthma, 80 million experiencing moderate-to-severe chronic obstructive pulmonary disease (COPD), and a significant number with mild COPD and other frequently undiagnosed CRDs [2]. According to the World Health Organization, CRDs were responsible for roughly 3 million fatalities in 2015, constituting 5% of all global deaths, with over 90% of these fatalities occurring in low- and middle-income nations [3].

In Africa, several asthma prevalence surveys have reported figures ranging from 4% to 14%, with an increasing trend in urban areas. The prevalence of COPD ranges from 4% to 25%, with higher rates in rural areas and older populations [4]. CRD prevalence in the general population is 1.8%, and 30% of outpatient consults in Morocco have respiratory symptoms [3]. In 85% of cases, patients have acute respiratory infections, and 15% have CRDs [5].

Several previous studies have shown that anxiety and depression are important comorbidities of chronic lung disease and are often associated with it [6]. Patients with CRDs are at risk of psychiatric disorders. The relationship between physiological parameters and psychological aspects lies in the interconnectedness of the body and mind. Psychological states and emotions often have a tangible impact on physiological functions, such as respiratory functions [7,8]. Studies have shown that psychiatric well-being influences lung function response and lung health [9]. A cross-sectional study of anxiety and depression in patients with CRDs revealed a high prevalence of anxiety and depression using the PRIME-MD (80%) [10]. Furthermore, according to the results of a 2014 survey in Egypt, 61.5% of a group of patients who suffered from CRDs had an anxiety disorder, and 58% had a depressive disorder [11]. To our knowledge, however, no studies have been published on anxiety and depression in Moroccan patients with CRDs.

The main objective of this study is to estimate the prevalence of depression and anxiety and identify their determinants in patients with CRDs in the Fès-Meknès region of Morocco. The aim is to take into account the psychological component in the management of the disease and to improve the mental health of these patients.

## Materials and methods

Study design and population

This cross-sectional study was conducted in the Pneumology Department of the Hassan II University Hospital in Fez, Morocco in 2021. Patients aged 18 years and older and diagnosed with CRDs who provided consent were recruited. Exclusion criteria encompassed patients who had verifiable intellectual disability, substantial psychopathological conditions before the diagnosis of respiratory disease, and/or significant neurocognitive disorders.

Sampling was done using the simple random approach, and the number of subjects required was calculated using the following formula: n = P (1-P).Z^2^α/i^2^, where n is the sample size, z is a 95% confidence level (z = 1.96), P (prevalence) of 13.65% represents the prevalence of anxiety in COPD patients in a Chinese study using the Hospital Anxiety and Depression Scale (HADS) scale [12], and i is precision equal to 5%. The sample size was calculated at 182 subjects.

Data collection

After obtaining approval from the Hassan II University Hospital Committee (approval number: 26/18), all subjects were informed of the conditions of the study and gave their written informed consent. The anonymity and confidentiality of all participants were ensured. Data were collected using a predefined questionnaire that elicited sociodemographic variables such as age, sex, residence, marital status, education level, occupation, monthly income, smoking status, alcohol consumption, and risk factors. Additionally, clinical variables were also noted such as medical and surgical history, clinical signs, and treatment.

Anxiety and depression were measured using the Moroccan version of the HADS [13]. In 1983, Zigmond and Snaith developed HADS as a tool to detect anxiety and depressive disorders in patients hospitalized in non-psychiatric environments, but it was subsequently validated for outpatient use. To identify anxiety and depressive disorders, this self-report scale comprises 14 items, with seven dedicated to the anxiety dimension and another seven to the depressive dimension. Each item elicits a response scored on a scale from 0 to 3, signifying the symptom’s intensity over the past week. The potential score range for each subscale spans from 0 to 21, where higher scores reflect the presence of more pronounced symptoms. Cutoff values were established for each subscale (anxiety and depression), with a score ranging from 0 to 7 classified as normal, whereas a score of 8 or greater indicated a significant disorder [14,15].

Statistical analysis

The descriptive statistics technique was used for the description of clinical, socioeconomic, and demographic variables such as age, place of residence, and date of diagnosis. We analyzed the association of depression and anxiety with sociodemographic and clinical variables using the t-test and the chi-square test. A p-value ≤0.05 was considered significant. All analyses were performed using the SPSS software version 26 (IBM Corp., Armonk, NY, USA).

## Results

Sociodemographic characteristics

A total of 209 subjects were included in the study. The mean age was 57.8 years (SD = 15.3). Approximately half (50.7%; n = 106) were female, and three-quarters (74.3%; n = 139) were married. Just over half (53.8%; n = 93) were illiterate, and most (73.8%; n = 127) were unemployed or housewives. Slightly over half (53.8%; n = 93) were covered by RAMED insurance, approximately half (51.2%; n = 105) came from rural areas, and most had never smoked (62.7%; n = 131). The sociodemographic characteristics are shown in Table 1.

**Table 1:** Sociodemographic characteristics.

Variable	n (%)
Age, mean ± SD (N = 209)	57.8 ± 15.3
Gender (N = 209)	
Male	103 (49.3)
Female	106 (50.7)
Habitat (N = 205)	
Rural	105 (51.2)
Urban	100 (48.8)
Marital status (N = 187)	
Married	139 (74.3)
Unmarried	48 (25.7)
Level of education (N = 173)	
Illiterate	93 (53.8)
Literate	80 (46.2)
Profession (N = 172)	
Unemployed	127 (73.8)
Employed	45 (26.2)
Lives (N = 185)	
Alone	5 (2.7)
With family	180 (97.3)
Smoking (N = 209)	
Non-smokers	131 (62.7)
Smokers/ex-smokers	78 (37.7)

Clinical characteristics

COPD was the most common pathology (43.1%; n = 90), followed by asthma (32.2%; n = 67). More than half (57.1%; n = 48) were obese or overweight. About half (50.8%; n = 178) had an associated comorbidity. The most frequent clinical symptom was dyspnea in 73.7% (n = 154) of patients, followed by cough in 56.6% (n = 118), asthenia in 44.0% (n = 92), and weight loss in 26.3% (n = 55). About a third (34.3%, n = 69) had desaturation on the six-minute walk test, 51.3% (n = 99) had an abnormal gasometry, and 14.4% (n = 30) were on oxygen therapy. Over two-thirds (71.3%; n = 149) of patients were on two or more treatments, and 93.8% (n = 196) had fewer than two hospitalizations per year. The clinical characteristics are presented in Table 2 and Table 3.

**Table 2:** Types of disease. COPD = chronic obstructive pulmonary disease

Variable	n (%)
COPD (N = 209)	
Yes	90 (43.1)
No	119 (56.9)
Severity group (N = 78)	
A or B	26 (33.3)
C or D	52 (66.7)
Level of control of COPD (N = 77)	
Well-controlled	36 (46.8)
Poorly controlled	41 (53.2)
Asthma (N = 208)	
Yes	67 (32.2)
No	141 (67.8)
Step (N = 56)	
1 or 2	10 (17.9)
3 or 4	46 (82.1)
Level of control of asthma (N = 56)	
Well-controlled	37 (66.1)
Poorly controlled	19 (33.9)
Diffuse interstitial lung diseases (N = 209)	
Yes	8 (3.8)
No	201 (96.2)
Bronchiectasis (N = 209)	
Yes	59 (28.2)
No	150 (71.8)
Sarcoidosis (N = 209)	
Yes	17 (8.1)
No	192 (91.9)
Sequelae disease (N = 208)	
Yes	32 (15.4)
No	176 (84.6)

**Table 3:** Clinical characteristics. BMI = body mass index; FEV1 = forced expiratory volume in one second

Variables	N (%)
Duration of disease, mean ± SD (N = 209)	7.5 ± 7.6
FEV1, mean ± SD (N=209)	1.5 ± 0.8
BMI (N = 86)	
BMI <18.5	11 (12.8)
18.5 ≤ BMI < 25	21 (24.4)
25 ≤ BMI < 30	18 (20.9)
BMI ≥30	36 (41.9)
Comorbidity (N = 178)	
High blood pressure	30 (16.8)
Diabetes	28 (15.7)
Cardiopathy	32 (17.9)
Nephropathy	10 (5.6)
Neoplasia	11 (6.2)
Other	67 (37.6)
surgical antecedents	43 (20.7)
Smoking (N = 209)	
Non-smokers	131 (62.7)
Smokers/ex-smokers	78 (37.7)
Cough	118 (56.5)
Dyspnea stage mMRC (N = 209)	
<2	99 (47.4)
≥2	110 (52.6)
Sputum	99 (47.6)
Wheezing	56 (26.8)
Pain	35 (16.7)
Hemoptysis	18 (8.6)
Infection	60 (28.7)
Fatigue	92 (44.0)
weight loss	55 (26.3)
Anorexia	41 (19.7)
Desaturation in the six-minute walk test	69 (34.3)
Respiratory insufficiency in gasometry	99 (51.3)
Patient under oxygen therapy	30 (14.4)
Number of treatments (N = 209)	
<2	60 (28.7)
≥2	149 (71.3)
Number of exacerbations (N = 209)	
<2/ year	138 (66.0)
≥2/ year	71 (34.0)
Number of hospitalizations (N = 209)	
<2/year	196 (93.8)
≥2/year	13 (6.2)

Depression

The prevalence of depression was 46.4% (n = 97) (95% CI = 39.6%, 53.2%), and the highest frequency was noted in patients with sarcoidosis (58.8%). In the bivariate analysis, depression was associated with the presence of dyspnea (51.3% vs. 32.7%; p = 0.018), presence of asthenia (56.5% vs. 38.5%; p = 0.009), use of oxygen therapy (66.7% vs. 42.7%; p = 0.015), higher number of hospitalizations (76.9% vs. 44.4%; p = 0.023), presence of anxiety (87.6% vs. 31.3%; p < 0.001), presence of COPD (55.6% vs. 39.5%; p = 0.021), and presence of asthma (35.8% vs. 51.8%; p = 0.031). We found no statistically significant association between depression and other variables (demographic, clinical, and therapeutic). The results for depression are shown in Table 4.

**Table 4:** Factors associated with Depression: results of the bivariate analysis. COPD = chronic obstructive pulmonary disease

Variables	Depression		P-value
	Yes, n (%)	No, n (%)	
Duration of disease (mean ± SD)	5.9 ± 5.3	8.6 ± 8.86	0.022
Age (mean ± SD)	59.4 ± 13.5	56.5 ± 16.7	0.186
Gender			
Female	48 (45.3)	58 (54.7)	0.740
Male	49 (47.6)	54 (52.4)	
Habitat			
Rural	52 (49.5)	53 (50.5)	0.428
Urban	44 (44.0)	56 (56.0)	
Marital status			
Married	61 (43.9)	78 (56.1)	0.124
Unmarried	15 (31.3)	33 (68.8)	
Level of education			
Illiterate	41 (44.1)	52 (55.9)	0.587
Literate	32 (40.0)	48 (60.0)	
Surgical antecedents			
No	81 (49.1)	84 (50.9)	0.096
Yes	15 (34.9)	28 (65.1)	
COPD			
No	47 (39.5)	72 (60.5)	0.021
Yes	50 (55.6)	40 (44.4)	
Asthma			
No	73 (51.8)	68 (48.2)	0.031
Yes	24 (35.8)	43 (64.2)	
Diffuse interstitial lung diseases			
No	95 (47.3)	106 (52.7)	0.290
Yes	2 (25.0)	6 (75.0)	
Bronchiectasis			
No	75 (50.0)	75 (50.5)	0.097
Yes	22 (37.3)	37 (62.7)	
Sarcoidosis			
No	87 (45.3)	105 (54.7)	0.284
Yes	10 (58.8)	7 (41.2)	
Sequelae disease			
No	80 (45.5)	96 (54.5)	0.424
Yes	17 (53.1)	15 (46.9)	
Pain			
No	76 (43.7)	98 (56.3)	0.077
Yes	21 (60.0)	14 (40.0)	
Dyspnea (mMRC)			
<2	18 (32.7)	37 (67.3)	0.018
≥2	79 (51.3)	75 (48.7)	
Fatigue			
No	45 (38.5)	72 (61.5)	0.009
Yes	52 (56.5)	40 (43.5)	
Anorexia			
No	72 (43.1)	95 (56.9)	0.076
Yes	24 (58.5)	17 (41.5)	
Gasometry			
No respiratory insufficiency	50 (33.2)	44 (46.8)	0.224
Respiratory insufficiency	44 (44.4)	55 (55.6)	
Number of hospitalizations			
<2/year	87 (44.4)	109 (55.6)	0.023
≥2/year	10 (76.9)	3 (23.1)	
Patient under oxygen therapy			
No	76 (42.7)	102 (57.3)	0.015
Yes	20 (66.7)	10 (33.3)	

Anxiety

The prevalence of anxiety was 57.4% (n = 120) (95% CI= 50.7%, 64.1%), and the highest frequency was noted in patients with diffuse interstitial lung diseases (75.0%). In the bivariate analysis, anxiety was associated with a history of surgery (37.2% vs. 62.4%; p = 0.003) and with the presence of COPD (66.7% vs. 50.4%; p = 0.019). We found no statistically significant association between anxiety and other variables (demographic, clinical, and therapeutic). The results for anxiety are shown in Table 5.

**Table 5:** Factors associated with anxiety: results of the bivariate analysis. COPD = chronic obstructive pulmonary disease

Variables	Anxiety		P-value
	Yes, n (%)	No, n (%)	
Duration of disease (mean ± SD)	6.0 ± 6.3	9.2 ± 8.6	0.004
Age (mean ± SD)	58.4 ±15.3	57.1 ± 15.4	0.534
Gender			
Female	60 (56.6)	46 (43.4)	0.810
Male	60 (58.3)	43 (41.7)	
Habitat			
Rural	63 (60.0)	42 (40.0)	0.469
Urban	55 (55.0)	45 (45.0)	
Marital status			
Married	26 (54.2)	22 (45.8)	0.911
Unmarried	74 (53.2)	65 (46.8)	
Level of education			
Illiterate	49 (52.7)	44 (47.3)	0.889
Literate	43 (53.8)	37 (46.3)	
Surgical antecedents			
No	103 (62.4)	62 (37.6)	0.003
Yes	16 (37.2)	27 (62.8)	
COPD			
No	60 (50.4)	59 (49.6)	0.019
Yes	60 (66.7)	30 (33.3)	
Asthma			
No	86 (61.0)	55 (39.0)	0.162
Yes	34 (50.7)	33 (49.3)	
Diffuse interstitial lung diseases			
No	115 (57.2)	86 (42.8)	0.767
Yes	5 (62.5)	3 (37.5)	
Bronchiectasis			
No	90 (60.0)	60 (40.0)	0.228
Yes	30 (50.8)	29 (49.2)	
Sarcoidosis			
No	112 (58.3)	80 (41.7)	0.368
Yes	8 (47.1)	9 (52.9)	
Sequelae disease			
No	102 (58.0)	74 (42.0)	0.612
Yes	17 (53.1)	15 (46.9)	
Pain			
No	99 (56.9)	75 (43.1)	0.735
Yes	21 (60.0)	14 (40.0)	
Dyspnea (mMRC)			
<2	62 (62.6)	37 (37.4)	0.148
≥2	58 (52.7)	52 (47.3)	
Fatigue			
No	63 (53.8)	54 (46.2)	0.239
Yes	57 (62.0)	35 (38.0)	
Anorexia			
No	92 (55.1)	75 (44.9)	0.212
Yes	27 (65.9)	14 (34.1)	
Gasometry			
No respiratory insufficiency	55 (58.5)	39 (41.5)	0.486
Respiratory insufficiency	53 (53.5)	46 (46.5)	
Number of hospitalizations			
<2/year	111 (56.6)	85 (43.4)	0.374
≥2/year	9 (69.2)	4 (30.8)	
Patient under oxygen therapy			
No	97 (54.5)	81 (45.5)	0.054
Yes	22 (73.3)	8 (26.7)	

## Discussion

The main objective of this study was to estimate the prevalence of depression and anxiety and identify their determinants in patients with CRDs in the Fès-Meknès region of Morocco. The prevalence of depression and anxiety was estimated at 46.4% and 57.4%, respectively, compared to a prevalence reported by a study in China of 13.65% and 10.79%, respectively, with 7.08% suffering from both [12].

Most patients in our study were at an advanced stage of the disease: 66.7% had COPD group C or D, and 82.1% had asthma stage 3 or 4. In a study in South Korea among 209 patients, the prevalence of clinical depression among patients at stable stages of the disease ranged from 10% to 42%, and the prevalence of anxiety ranged from 10% to 19%. In patients with advanced disease, the prevalence of depression and anxiety was higher, ranging from 37% to 71% and 51% to 75%, respectively, as measured by the Korean version of the HADS [16].

The results of our study revealed a high prevalence of depression and anxiety in patients with COPD (55.6% and 66.7%, respectively). These results agree with the study conducted by Halimi, which found a high prevalence of depression and anxiety (from 31% to 71% and 51% to 75%, respectively) in patients with COPD. Moreover, the prevalence of depression and anxiety among asthmatic patients was 35.8% and 50.7%, respectively. These findings align with the study by Halimi regarding the prevalence of depression, which revealed rates between 25% and 50% for depression in asthmatic patients. However, there was a contrast in terms of anxiety, which was higher in our study (50.7%) compared to the 20-35% range in the Halimi study [17].

Our study showed a high prevalence of anxiety and depression in patients with CRDs. This higher prevalence can be explained by the delay in diagnosis and the lack of communication and social support. It could also be related to the crisis following COVID-19, as our study was conducted after the pandemic. Thus, a fear of infection by this virus would have made the respiratory manifestations more painful and more difficult to live with.

The association between depressive and anxiety disorders and clinical signs is not consistent across studies. In our study, symptoms such as dyspnea and fatigue, duration of disease, the use of oxygen therapy, and the number of hospitalizations were associated with depression, which is consistent with the results of several other studies [15,18,19]. This finding is in contrast to that of Lee et al., who showed that clinical signs and number of hospitalizations had no effect on the incidence of depression [16]. Anxiety, however, was only associated with the duration of illness and previous surgery, which can be explained by the initial absence of knowledge of the disease. The anxiety experienced by individuals could be influenced by the prolonged experience of dealing with the illness over time and the impact of past surgeries, potentially due to the initial unfamiliarity or lack of information regarding their medical condition.

Our study is the first to determine the prevalence of anxiety and depression among Moroccan patients suffering from CRDs. It has highlighted a significant prevalence of depression and anxiety among patients with CRDs. Further investigations are needed to better understand these diseases, their comorbidities, and the factors associated with depression and anxiety in these patients. Additionally, it advocates for raising awareness among physicians about the psychiatric implications of these diseases and suggests using an early systematic screening tool for psychiatric disorders in the follow-up of patients with CRDs. Furthermore, it provides a basis for further in-depth research and positive practical implications, especially within this population.

Finally, these results should be interpreted with the methodological limitations of cross-sectional studies. First, the study population does not reflect all patients with CRDs, especially those in the private sector, who may not have the same sociodemographic and clinical characteristics. Second, some patients refused to answer the questionnaire.

## Conclusions

The results of this study have shown a high prevalence of depression and anxiety in patients with CRDs. Therefore, we recommend that future research advances the understanding of the diseases and their comorbidities. Medical practitioners should adopt a tool for early systematic screening of psychiatric disorders in the follow-up of patients with CRDs. Further, all general practitioners and specialists should be educated on the importance of psychiatric complications after CRDs. Finally, more studies should be conducted to describe the predictors of depression and anxiety in patients with CRDs in our population.
